# Molecular basis determining species specificity for TLR2 inhibition by staphylococcal superantigen-like protein 3 (SSL3)

**DOI:** 10.1186/s13567-018-0609-8

**Published:** 2018-11-28

**Authors:** Kirsten J. Koymans, Louris J. Feitsma, Adinda Bisschop, Eric G. Huizinga, Jos A. G. van Strijp, Carla J. C. de Haas, Alex J. McCarthy

**Affiliations:** 1 Department of Medical Microbiology, University Medical Center Utrecht, Utrecht University, Utrecht, The Netherlands; 2 Crystal and Structural Chemistry, Department of Chemistry, Faculty of Science, Bijvoet Center for Biomolecular Research, Utrecht University, Utrecht, The Netherlands

## Abstract

*Staphylococcus aureus* is a versatile opportunistic pathogen, causing disease in human and animal species. Its pathogenicity is linked to the ability of *S. aureus* to secrete immunomodulatory molecules. These evasion proteins bind to host receptors or their ligands, resulting in inhibitory effects through high affinity protein–protein interactions. Staphylococcal evasion molecules are often species-specific due to differences in host target proteins between species. We recently solved the crystal structure of murine TLR2 in complex with immunomodulatory molecule staphylococcal superantigen-like protein 3 (SSL3), which revealed the essential residues within SSL3 for TLR2 inhibition. In this study we aimed to investigate the molecular basis of the interaction on the TLR2 side. The SSL3 binding region on murine TLR2 was compared to that of other species through sequence alignment and homology modeling, which identified interspecies differences. To examine whether this resulted in altered SSL3 activity on the corresponding TLR2s, bovine, equine, human, and murine TLR2 were stably expressed in HEK293T cells and the ability of SSL3 to inhibit TLR2 was assessed. We found that SSL3 was unable to inhibit bovine TLR2. Subsequent loss and gain of function mutagenesis showed that the lack of inhibition is explained by the absence of two tyrosine residues in bovine TLR2 that play a prominent role in the SSL3–TLR2 interface. We found no evidence for the existence of allelic SSL3 variants that have adapted to the bovine host. Thus, within this paper we reveal the molecular determinants of the TLR2–SSL3 interaction which adds to our understanding of staphylococcal host specificity.

**Electronic supplementary material:**

The online version of this article (10.1186/s13567-018-0609-8) contains supplementary material, which is available to authorized users.

## Introduction

*Staphylococcus aureus* is a human and animal commensal and pathogen and characterized by its ability to secrete a wide range of virulence factors, including immune evasion molecules [1, 2]. These immune evasion molecules are proteins that interfere with distinct parts of the immune system. Most inhibitory effects are mediated through protein–protein interactions, in which the secreted staphylococcal protein binds to a host protein (enzyme or receptor) and thereby inhibits its function [2]. These protein–protein interactions are often highly specific, possess very high affinities (in the nanomolar range), and generally involve only a limited number of amino acid residues.

High-resolution structural data can assist in the identification of binding interfaces between immune evasion and host molecules. This data gives a highly accurate view of the interaction sites, and can thereby identify direct amino acid interactions. Subsequent analysis of mutant proteins enables identification of the amino acids contributing most to binding affinity, which can come down to one or two critical residues. Due to these highly specific interactions, many of the evasion molecules of *S. aureus* are species-specific or at least have a limited species range [2]. This can either be related to differential expression of host receptors that are targeted, or which is most often the case, by variations within the host receptors themselves.

The staphylococcal superantigen-like proteins (SSLs) are a family of structurally related proteins consisting of a total of 14 members and are involved in immune evasion. They all contain an N-terminal oligomer-binding fold (OB-fold) and a C-terminal β-grasp domain, which are both common folds seen in many staphylococcal evasion proteins [2, 3]. Despite their structural similarities, these proteins have a diverse range of interaction partners. For instance, SSL1 and SSL5 bind to and inhibit matrix metalloproteinases [4, 5], SSL3 and SSL4 inhibit toll-like receptor 2 (TLR2) signaling [6–8], and SSL5 and SSL11 are both able to bind and inhibit the functions of several glycoproteins, including PSGL-1 [9, 10]. The fact that the SSLs can bind to a broad range of targets is related to the stable fold of the SSL proteins that allows for large variations in amino acid sequences which enables binding to distinct protein partners [2]. Indeed, amino acid identity between SSLs is typically only about 40%. The SSL family of proteins is located on the core variable genome of *S. aureus*, divided over two clusters: pathogenicity island vSaα encodes SSL1–SSL11 and immune evasion cluster 2 (IEC2) encodes for SSL12–SSL14 [2]. Generally speaking, these core variable genome encoded evasion molecules have a more broad specificity than the evasion molecules that are encoded on mobile genetic elements, such as CHIPS and SCIN, that are often found in strains associated with a specific target host [2]. Indeed the SSL proteins are generally not human specific and often also able to bind and inhibit the murine counterparts, making in vivo studies feasible. This has already been demonstrated for SSL3, SSL5, SSL7, and SSL10 [11–15].

TLR2 is an important immune receptor that recognizes pathogen-associated molecular patterns (PAMPs) present on bacterial pathogens and thereby assists in bacterial clearance [16]. TLR2 binds bacterial di- and triacylated lipopeptides through heterodimerization with TLR6 and TLR1, respectively [17, 18]. Both ligand binding and receptor dimerization are crucial for further signaling through MyD88 and NK-κB, which results in the initiation of immune responses, including cytokine productions [16]. TLR2 is important in the defense against *S. aureus* and recognizes lipoproteins present on the bacteria [19]. It is therefore not surprising that *S. aureus* secretes TLR2 inhibitors, SSL3 and SSL4, with SSL3 being more potent than SSL4, to prevent recognition by the immune system. We recently solved the crystal structure of the murine TLR2–SSL3 complex and elucidated the inhibitory mechanism of TLR2 inhibition by SSL3 [8]. We found that SSL3 inhibits TLR2 in a dual mechanism, by interfering with lipopeptide binding as well as with heterodimerization of TLR2 with TLR1 or TLR6; processes that are vital for TLR2 downstream signaling and immune function. The crystal structure revealed that SSL3 and TLR2 make contact through a highly hydrophobic interface and identified the amino acids most likely to contribute most to binding [8]. Through subsequent investigation of loss of function mutants of SSL3, we revealed the exact amino acids in SSL3 that are required for the interaction with TLR2. Seven residues in SSL3 were shown to be involved, with a key role for two clustered phenylalanine residues, Phe156 and Phe158 [8]. The difference in TLR2 inhibitory activity between SSL3 and SSL4 could also be fully attributed to differences in these specific residues. On the TLR2 side, however, the crucial amino acids required for the SSL3 interaction remain to be determined. In order to improve understanding of the molecular basis of the SSL3–TLR2 interaction we have investigated the interaction interface on the TLR2 side. First, we compared the TLR2s from different animal species and found several interspecies differences in TLR2 in the SSL3–TLR2 binding region. We determined that these differences alter the SSL3 inhibitory potential in different species and show that bovine TLR2 signaling is not inhibited by SSL3. Through the analysis of loss and gain of function TLR2 mutants, we managed to pinpoint the most critical amino acids for interaction, two tyrosine residues in TLR2, Y326 and Y376. Furthermore, we have examined the presence and activity of allelic *ssl3* variants within bovine strains to study host adaptation, but found no evidence for the existence of host-adapted SSL3 variants. Altogether, this study adds to our understanding of the molecular interaction between SSL3 and TLR2 and explains the species specificity of SSL3.

## Materials and methods

### Proteins and reagents

SSL3 from strain NCTC 8325 (full length protein and truncated protein comprising residues 134–326) and from bovine strains CC771 and CC97 (full length protein) and SSL4 from strain NCTC 8325 (full length and truncated protein comprising residues 79–278) and from bovine strain CC97 (full length protein) were cloned, expressed, and isolated as previously described [8, 9]. In short, SSL3 and SSL4 variants were expressed with an N-terminal His_6_-tag in *Escherichia coli* Rosetta-gami(DE3)pLysS, after which proteins were isolated and stored in PBS and protein purity was determined to be > 95% by SDS-PAGE. The TLR2 agonist MALP-2 was purchased from Santa Cruz, and the agonists Pam_2_CSK_4_ and Pam_3_CSK_4_ were purchased from EMC microcollections. The human IL-8 ELISA kit was purchased from Sanquin.

### Cloning and expression of TLR2

We cloned human (NM_003264.3), bovine (AF368419.1), mouse (NM_011905.3), and equine (NC_009145.2) TLR2 and replaced each signal peptide (human aa1–20, bovine aa1–20, mouse aa1–24, and equine 1–20) by the PreProTrypsin signal peptide (MSALLILALVGAAVA), adapted from the pFLAG-CMV-1 vector (Sigma). To create loss of function mutants within human TLR2, we mutated the following tyrosine residues to the corresponding amino acids present in bovine TLR2: Y323F, Y326H, and Y376T. For bovine TLR2 gain of function mutants we generated F323Y, H326Y, and T376Y mutants. Mutations were constructed via overlap PCR. All combinations of single, double, and triple mutants were constructed within both human and bovine TLR2. To enable proper detection, we attached an N-terminal FLAG tag (DYKDDDDK) followed by a flexible linker (GGS) to the N-terminus of each TLR2. For stable expression of TLR2 in HEK293T cells, we used a lentiviral expression system. To this end, we cloned the TLR2 constructs (PreProTrypsin signal peptide-FLAG-GGS-TLR2), preceded by a KOZAK sequence, in a dual promoter lentiviral vector, derived from no. 2025.pCCLsin.PPT.pA.CTE.4x-scrT.eGFP.mCMV.hPGK.NGFR.pre, kindly provided by Dr Luigi Naldini (San Raffaele Scientific Institute, Milan, Italy), as described previously [20]. This altered lentiviral vector (BIC-PGK-Zeo-T2a-mAmetrine; EF1A) uses the human EF1A promoter to facilitate potent expression in immune cells and expresses the fluorescent protein mAmetrine and selection marker ZeoR. Virus was produced in 24-well plates using standard lentiviral production protocols and the third-generation packaging vectors pMD2G-VSVg, pRSV-REV, and pMDL/RRE. Briefly, 0.25 µg lentiviral vector and 0.25 µg packaging vectors were cotransfected in HEK293T cells by using 1.5 µL Mirus LT1 transfection reagent (Sopachem, Ochten, The Netherlands). After 72 h, 100 µL unconcentrated viral supernatant adjusted to 8 µg/mL polybrene was used to infect ~ 50 000 HEK293T cells by spin infection at 1000 g for 2 h at 33 °C. HEK293T cells were selected with 500 µg/mL zeocin for TLR2 expression. HEK293T cells were obtained from ATCC (American Type Culture Collection) and grown in DMEM medium supplemented with penicillin/streptomycin and 10% FCS. For FLAG-tag detection, HEK-TLR2 cells were detached using PBS/3 mM EDTA and washed in RPMI, containing 0.05% human serum albumin (RPMI/HSA). HEK-TLR2 cells (5 × 10^6^/mL) were stained with 10 µg/mL anti-FLAG M2 mAb (Sigma), washed with RPMI/HSA and subsequently stained with phycoerythrin-labeled goat-anti-mouse Ig before FLAG-tag detection on a flow cytometer (FACSVerse, BD Biosciences).

### HEK-TLR2 stimulation

HEK-TLR2 cells were seeded in 96-well plates in 100 µL at a concentration of 2.5 × 10^5^ cells/mL. The day after, HEK-TLR2 cells were in some cases preincubated with 10 µL of HIS-SSL3 (final concentration of 10 µg/mL) or HIS-SSL4 (final concentration ranging from 30 to 1 µg/mL) for 30 min at 37 °C, before stimulation with 10 µL of one of the different TLR2 agonists: MALP-2 (final concentrations ranging from 300 to 0.005 ng/mL) and Pam_2_CSK_4_ (final concentrations ranging from 300 to 0.0005 ng/mL) or Pam_3_CSK_4_ (final concentrations ranging from 30 to 0.005 µg/mL). After 6 h of TLR2 stimulation, cell free supernatants were collected for detection of IL-8 production by ELISA (Sanquin), following manufacturer’s protocol. Optical densities were measured in a plate reader at OD_450_. For the stimulation experiments shown in Figure 3 and in Figure 4 truncated SSL3 (residues 134–326) was used. For stimulations in Figure 5 full length SSL3 (residues 1–326) was used to include the possibility of host adaptation through a distinct mechanism found in the SSL3 N-terminus. Similarly, for SSL4 truncated protein (residues 79–278) was used in Additional file 1 and full length protein (residues 1–278) was used in Figure 6.

### SSL3 binding experiments

To determine SSL3 binding to the different HEK cell lines, cells were treated with trypsin (it was confirmed that trypsin treatment did not affect the assay), harvested, and in some cases first pretreated for 45 min with neuraminidase (0.2 U/mL, from *Clostridium perfringens*, Roche) at 37 °C to remove sialic acid residues. Afterwards, cells were allowed to bind to different concentrations of HIS-SSL3 (variant 134–326, final concentrations ranging from 10 to 0.1 µg/mL) for 30 min on ice before addition of an anti-HIS-FITC monoclonal antibody (1:20, LS Biosciences). Binding was measured on a flow cytometer.

### Alignments and sequencing

**Figure 1:**
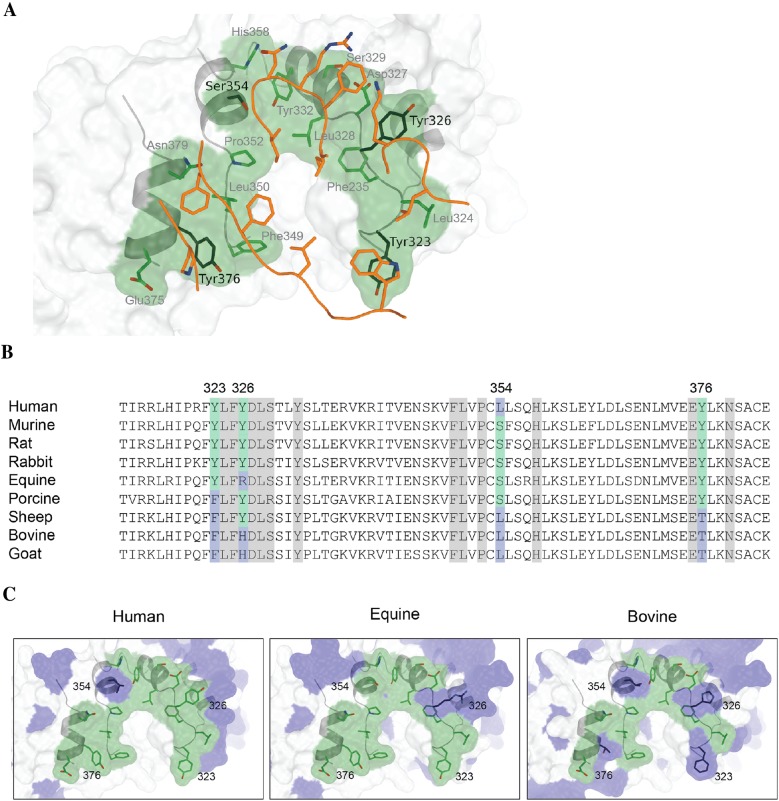
**The SSL3–TLR2 binding interface reveals differences in species within the SSL3 binding region of TLR2. A** The binding interface between SSL3 and murine TLR2 revealed 16 potential sites of interaction between SSL3 and TLR2. SSL3 is shown in orange and TLR2 is shown in green. Image adapted from Koymans et al. [8] (PDB ID code 5D3I). **B** Sequence alignment of the TLR2 region involved in SSL3 binding of nine different animal species. Conserved amino acids found in the binding interface are highlighted in gray, while the amino acids that differ between species are highlighted in green (conserved as compared to murine TLR2) or blue (different as compared to murine TLR2). **C** Homology models of human, equine, and bovine TLR2 derived from the structure of the murine TLR2–SSL3 complex (PDB ID code 5D3I). TLR2 is shown in green and the amino acids and regions that differ have been highlighted in blue.

Alignment of the different TLR2s shown in Figure 1B were performed using Clustal Omega analysis. For numbering of amino acid residues the human/murine TLR2 has been used as a reference point. Residue numbers in other species may differ, depending on their relative position (e.g. for bovine TLR2 all mentioned residues are +1).

Sequences used for Figures 5, 6 and Additional files 3, 4 were previously aligned in McCarthy and Lindsay [21]. The sequences of *ssl3* genes from additional bovine isolates provided by Jodi Lindsay (St George’s University of London, London, UK) (CC130 C01611, CC771 32932, CC771 30425, CC771 32933, CC97 C01899, CC97 C01312, CC151 C123/5/05-29, CC151 C123/5/05-22, CC151 982BL, CC188 30375, CC188 30296, CC188 818) were amplified (Forward primer 5′-GTGATTATCTTAGAACGCCATC-3′ and Reverse primer 5′- GAAGCTAAGCAACATGTAAAC-3′, thermocycling conditions), sequenced (Macrogen) and included in the alignment. The maximum likelihood trees (Figures 5 and 6) were produced in MEGA v6.05 with JTT model of amino acid substitutions and 2000 bootstraps. Numbers on the tree represent bootstrap support values with a value of 1.00 = 100% confidence in that branching event. *ssl3* and *ssl4* sequence alignments were tested for conforming to a codon-based test of neutrality in MEGA v6.05.

### Homology modelling

Homology models of human (Uniprot-ID O60603), equine (Q6T752), and bovine TLR2 (Q95LA9) were created using the ProtMod modeling package from the Fold and Function Assignment System (FFAS) server [22]. Models were built using the SCWRL-algorithm [23] and the structure of mouse TLR2 from the SSL3–TLR2 complex (PDB-ID: 5D3I) [8] as a template supplemented with a sequence alignment between the target species and the corresponding region of murine TLR2 (res. 25–589; Q9QUN7).

### Statistical analysis

Statistical analysis was performed with GraphPad Prism using a one-way ANOVA followed by either Sidak’s (Figure 4) or Tukey’s (Figures 5, 6) multiple comparisons test to compare between groups. For Figure 4, the mean of the control group (either hTLR2 for Figure 4C or bTLR2 for Figure 4D) was compared to all columns and the TLR2 single mutants that showed altered SSL3 inhibitory activity were compared to all subsequent double and triple mutants.

## Results

### Defining differences in the SSL3–TLR2 binding interface between species

Using the crystal structure of the SSL3–TLR2 complex, a total of 16 amino acids were identified in murine TLR2 that could be involved in formation of the specific inhibitory complex (Figure 1A). To investigate the species specificity of the interaction we determined whether there are differences in the TLR2–SSL3 binding interface between different animal species by sequence alignment and homology modeling. We aligned TLR2 sequences from nine different species, including many natural hosts of *S. aureus* (Figure 1B). The overall homology of the TLR2s (on amino acid level) varies from 71 to 82% as compared to human TLR2. In the SSL3–TLR2 binding interface (residue 323–379) the overall homology is higher, varying from 79 to 91%. The homology in this region is likely to be higher because it is also involved in ligand binding and heterodimerization with TLR1 and TLR6 [17, 18].

The murine TLR2-surface in the SSL3-murine TLR2 complex that likely promotes stable binding of SSL3 was analyzed. Murine TLR2-residues proximate to SSL3 (within 5 Å) and residues at homologous positions in the TLR2s from other species are highlighted in the alignment. All amino acids that are conserved between the species are shown in light gray, whereas amino acids that differ are highlighted in green or blue (Figure 1B, using murine TLR2 as the reference sequence, three tyrosine residues Tyr323, Tyr326, and Tyr376 and one serine residue Ser354). Murine, rat, and rabbit TLR2 share all amino acids in the SSL3 binding interface. Human, equine and porcine TLR2 differ at one position (Ser354, Tyr326 and Tyr323, respectively) compared to murine TLR2, sheep TLR2 differs at Tyr323, Ser354, and Tyr376, and goat and bovine TLR2 differ most, namely in all three tyrosine residues  as well as the serine residue.

Homology modeling based on murine TLR2 of the interfaces of human, equine, and bovine TLR2 in complex with SSL3 visualized the differences (shown in blue) in the binding site (Figure 1C). Human and murine TLR2 are highly similar with the only difference being the serine residue that is located in the periphery of the binding interface. The modeling of equine TLR2 shows a stronger alteration, a long and positively charged arginine residue instead of the neutral and hydrophobic tyrosine residue that may be in a critical site of the SSL3–TLR2 interface. Bovine TLR2 differs extensively, containing two different hydrophobic residues (Phe instead of Tyr) at two of the positions (Tyr323 and Tyr326), which, however, are similar amino acids, both in hydrophobic nature and structure. At position 376 bovine TLR2 contains a Thr instead of Tyr, which has a smaller side chain offering substantially less surface for hydrophobic interaction and could alter the affinity of the complex markedly.

### SSL3 has species-specific activity and does not inhibit bovine TLR2

**Figure 2:**
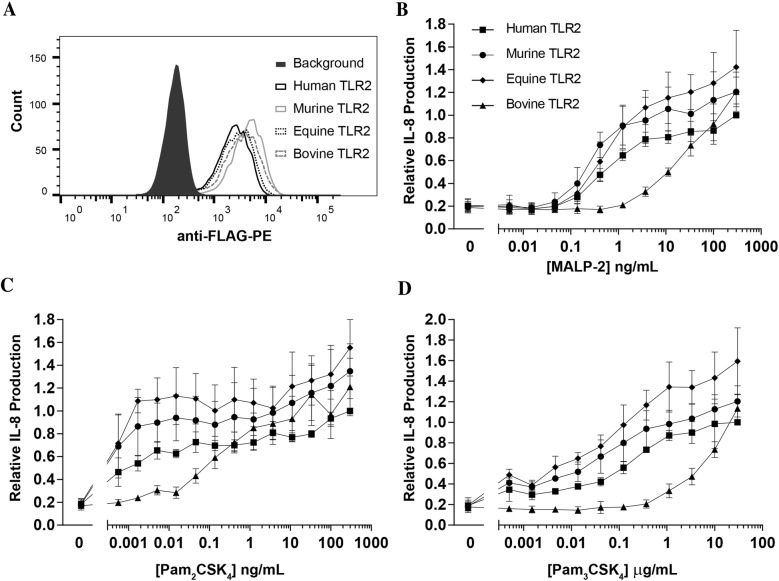
**Stimulation of different TLR2 receptors by three TLR2 agonists. A** All stable HEK293T cell lines express comparable amounts of FLAG-tagged TLR2, as detected by anti-FLAG staining using flow cytometry. One representative experiment is shown (**B**–**D**). All stable HEK293T-TLR2 expressing cell lines were stimulated with different concentrations of the TLR2 agonists MALP-2 (**B**), Pam_2_CSK_4_ (**C**), and Pam_3_CSK_4_ (**D**) for 6 h, after which supernatant was harvested and IL-8 production within the supernatant was determined using an anti-IL8 ELISA. The data shown represents the relative IL-8 production (IL-8 production was normalized to the highest agonist concentrations for human TLR2). The mean ± standard deviation (SD) of at least three independent experiments is shown.

To determine whether the identified differences in the binding interface also result in altered SSL3 function, we created stable HEK293T cell lines individually expressing bovine, equine, human, or murine full length TLR2 containing an N-terminal FLAG tag. All cell lines express comparable levels of TLR2 as determined by anti-FLAG staining (Figure 2A). Cell lines were stimulated with concentration ranges of three potent TLR2 agonists: the two diacylated lipopeptides MALP-2 (Figure 2B) and Pam_2_CSK_4_ (Figure 2C) and the triacylated lipopeptide Pam_3_CSK_4_ (Figure 2D). Since the HEK cell lines only contain endogenous levels of human TLR1 and TLR6 [24, 25], all TLR2s from different species are apparently able to signal using the human counterparts, although to different extents. Interestingly, bovine TLR2 was less potently activated by all three ligands and most noticeably by MALP-2 and Pam_3_CSK_4_. Pam_2_CSK_4_ showed a delay in response at low concentrations for bovine TLR2, but was capable of activating all TLR2s in a similar fashion at higher concentrations (Figure 2C). To examine and compare the inhibitory potential of SSL3 on the different receptors, agonist concentrations were chosen based on their ability to stimulate all TLR2 receptors most similarly and thus were in the higher range.

**Figure 3:**
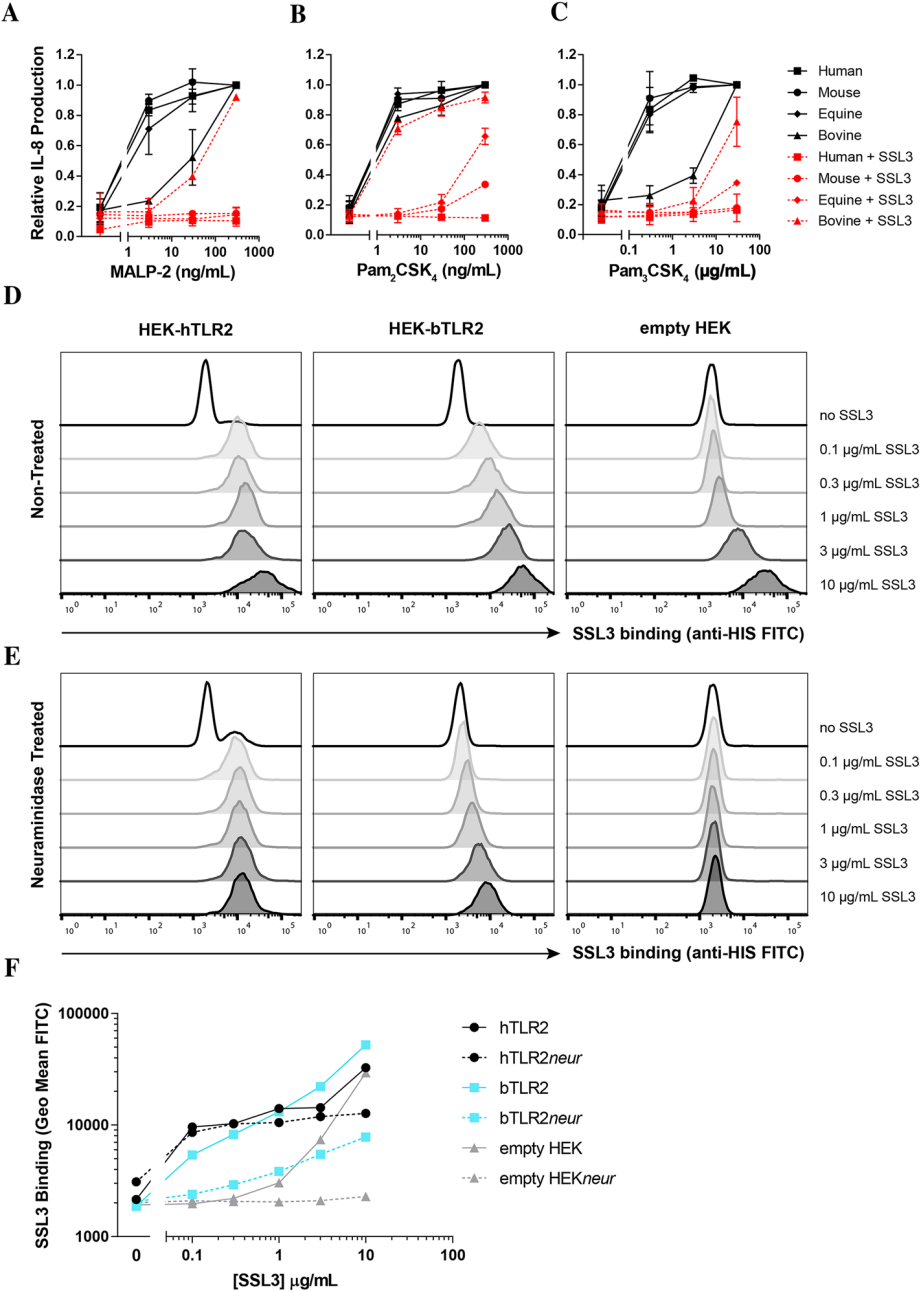
**SSL3 has a limited species range and does not inhibit bovine TLR2. A**–**C** Stable HEK293T cell lines expressing human, murine, equine, and bovine TLR2 were stimulated for 6 h with the three TLR2 agonists MALP-2 (**A**), Pam_2_CSK_4_ (**B**), and Pam_3_CSK_4_ (**C**), in the absence (black) or presence (red) of 10 μg/mL SSL3. Relative IL-8 production was determined by taking the maximum stimulus per species and all data points per species (with and without SSL3) were normalized to this point. Data points represent mean ± SD of two independent experiments. **D**–**F** SSL3 binding (final concentrations ranging from 10 to 0.1 μg/mL) to hTLR2, bTLR2, and empty HEK cells was examined without (**D**) or after neuraminidase treatment (**E**). HIS-SSL3 was allowed to bind on ice for 30 min before addition of an anti-HIS-FITC monoclonal antibody. Binding was measured using flow cytometry. **D**, **E** Histogram overlays of the original flow cytometer data. One representative experiment out of three independent experiments is shown. **F** Represents the geometric mean fluorescence of the FITC signal (SSL3 binding) of the same binding experiments shown in **D** and **E**. SSL3 binding to hTLR2 is shown in black, to bTLR2 in blue, and to empty HEK cells in gray. Solid lines represent the non-treated cell lines and dotted lines represent the cell lines after neuraminidase treatment.

Addition of SSL3 (from strain NCTC 8325) to the cells prior to agonist stimulation inhibited the stimulatory capacities of human TLR2, murine TLR2, and equine TLR2 (Figures 3A–C). Strikingly, bovine TLR2 signaling was not inhibited by SSL3 for all three agonists. Thus, SSL3 is not capable of inhibiting bovine TLR2. This is probably related to the differences seen in the binding interface between human and bovine TLR2 shown in Figure 1. SSL3 has a closely related family member, SSL4, that also inhibits TLR2 in the same manner as SSL3, although for human TLR2 it is a much less efficient inhibitor in comparison to SSL3 (100-fold less active) [6]. We examined the ability of SSL4 to inhibit bovine TLR2 and found that SSL4 was also not active on bovine TLR2 (Additional file 1). Thus, both SSL3 and SSL4 are active in a limited species range.

To complement the functional data we analyzed binding of SSL3 to the human and bovine TLR2 cell lines. We have previously shown that the SSL3–TLR2 interaction is purely based on protein–protein interactions and independent of glycan binding through the sialyl Lewis^X^ (sLe^X^) binding motif found in SSL3 [8]. This sugar binding motif is however involved in (non-specific) binding of SSL3 to cells [6, 7], which complicates SSL3–TLR2 binding studies due to high non-TLR2 dependent background binding. Indeed, when we examined binding of SSL3 to human TLR2-expressing, bovine TLR2-expressing, and empty HEK cells, non-specific binding to the empty HEK cells was detected, especially at higher SSL3 concentrations (Figure 3D shows the original FACS data and Figure 3F a summary). To prevent sLe^X^-mediated binding, HEK cells were treated with neuraminidase, which removes sialic acid residues, prior to assessment of SSL3 binding. Neuraminidase treatment of the TLR2 cell lines (Figures 3E and F) showed complete abrogation of SSL3 binding to empty HEKs, confirming that in the TLR2 cell lines specific SSL3–TLR2 interactions could now be examined. The neuraminidase treatment had almost no effect on binding of SSL3 to human TLR2, indicating a highly specific interaction of SSL3 with human TLR2. For bovine TLR2, the binding of SSL3 was greatly reduced by neuraminidase treatment, however residual binding could still be observed at higher SSL3 concentrations as compared to the empty HEK cells. This indicates that SSL3 can still bind bovine TLR2, although the binding is of much lower affinity than the binding to human TLR2, which therefore likely results in the incapability of SSL3 to inhibit bovine TLR2 signaling.

### Identification of critical TLR2-residues in the interface

The differences between bovine and human TLR2 revolve around the three earlier described tyrosine residues: Tyr323, Tyr326, and Tyr376 (Figure 1). To examine whether these three amino acids determine the species specific differences of SSL3 and its inability to inhibit bovine TLR2, we made mutants of both human and bovine TLR2 to create “loss of SSL3 inhibition” and “gain of SSL3 inhibition” mutants, respectively.

**Figure 4:**
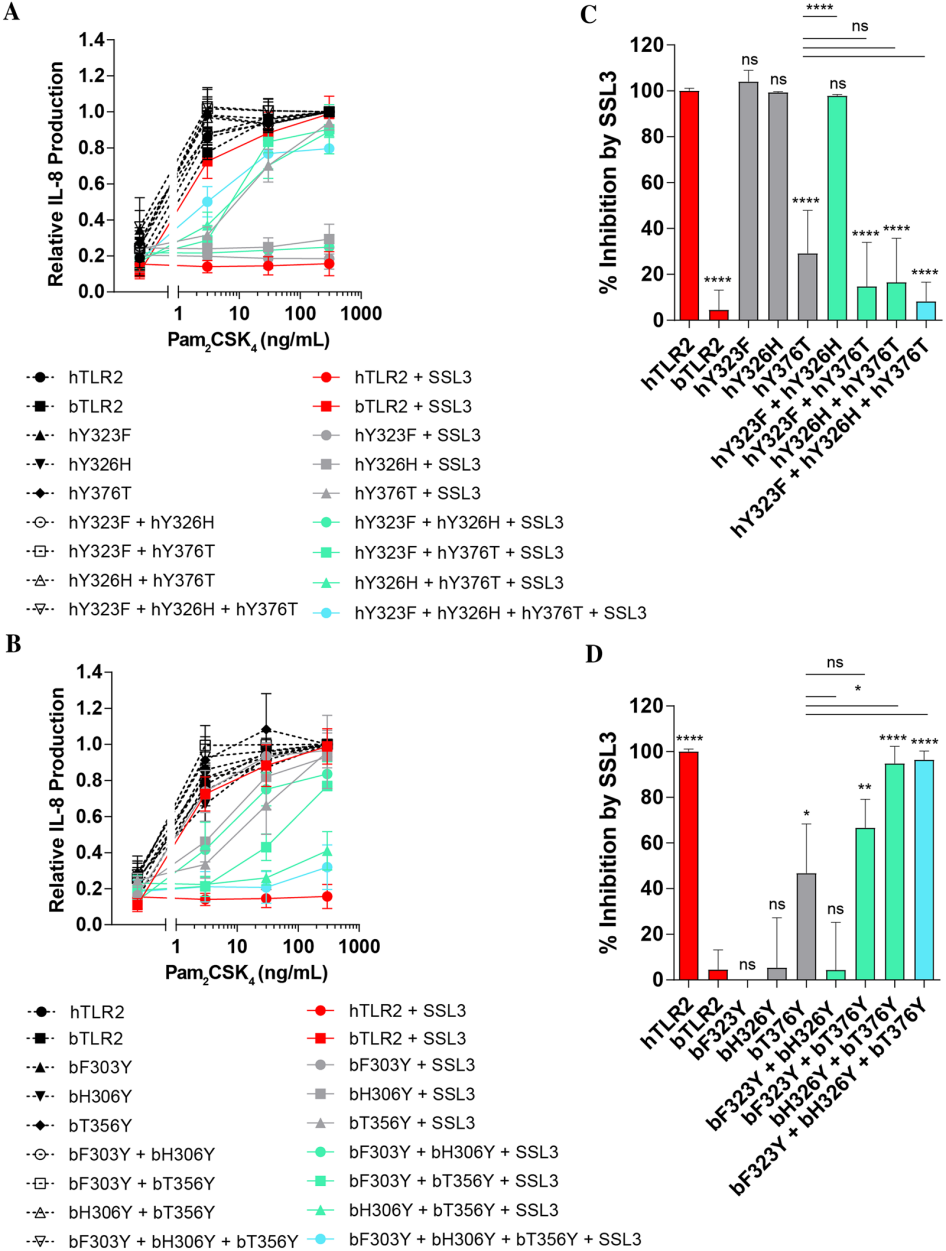
**Loss and gain of function mutants of human and bovine TLR2. A** Bovinized human TLR2 mutants and **B** humanized bovine TLR2 mutants. Stable HEK293T cell lines were created for all mutant TLR2s (wild-type in red, single mutants in gray, double mutants in green, and triple mutants in blue). Cell lines were stimulated for 6 h with a concentration range of Pam_2_CSK_4_ in presence or absence of SSL3. Relative IL-8 production was determined by taking the maximum stimulus for each cell line and all other data points per cell line (with and without SSL3) were normalized to this. **C**, **D** Percentage inhibition was calculated at a stimulus of 30 ng/mL Pam_2_CSK_4_ for human (**C**) and bovine (**D**) mutants. Data points represent the mean ± SD of at least three independent experiments. Statistical significance was determined by a one-way Anova. *p ≤ 0.05, **p ≤ 0.01, ****p ≤ 0.0001, ns (non-significant), followed by Sidak’s multiple comparisons test. All bars were compared to the control group (human TLR2 for **C** and bovine TLR2 for **D**) and if a significant difference was determined for a single mutant, this mutant was subsequently compared to all double or triple mutants.

First, the human TLR2 receptor was bovinized by replacing the human tyrosine residues by their respective bovine counterparts. Single, double, and triple mutants of all three tyrosine residues were created and stable HEK293T cell lines were generated for all mutants. Using anti-FLAG staining, expression was confirmed to be in a similar range (Additional file 2). Because Pam_2_CSK_4_ was the only stimulus tested that gave equal responses for human and bovine TLR2 we chose to use this ligand in all further experiments. We confirmed that all mutant cell lines were efficiently stimulated by Pam_2_CSK_4_ (Additional file 2). To determine the effects of bovinization of human TLR2 all cell lines were stimulated with Pam_2_CSK_4_ in presence or absence of SSL3 (Figures 4A and C). Single mutation of residue Tyr376 to Thr376 results in a dramatic loss of function of SSL3. The other two tyrosine residues do not show any effect, not even when combined. In comparison to the Tyr376Thr mutation, the triple loss of function mutant did appear to have reduced inhibition by SSL3, however this difference was not significant. Thus, it is clear that in these loss of function mutants, residue Tyr376 is the determining factor for SSL3 function. From our analysis of the gain of function mutants created by humanizing bovine TLR2, a similar picture emerged (Figure 4B, D). Again, among the three single mutants the Thr376Tyr mutation showed the largest gain of SSL3 function. However, the replacement His326Tyr also increased SSL3 inhibitory potential. Interestingly, only the combination of the two resulted in full inhibition of bovine TLR2 by SSL3. Thus, Tyr326 and Tyr376 are important residues involved in the SSL3–TLR2 interaction, with a major role for Tyr376.

### SSL3 is conserved in strains of bovine origin and shows no sign of host adaptation

**Figure 5:**
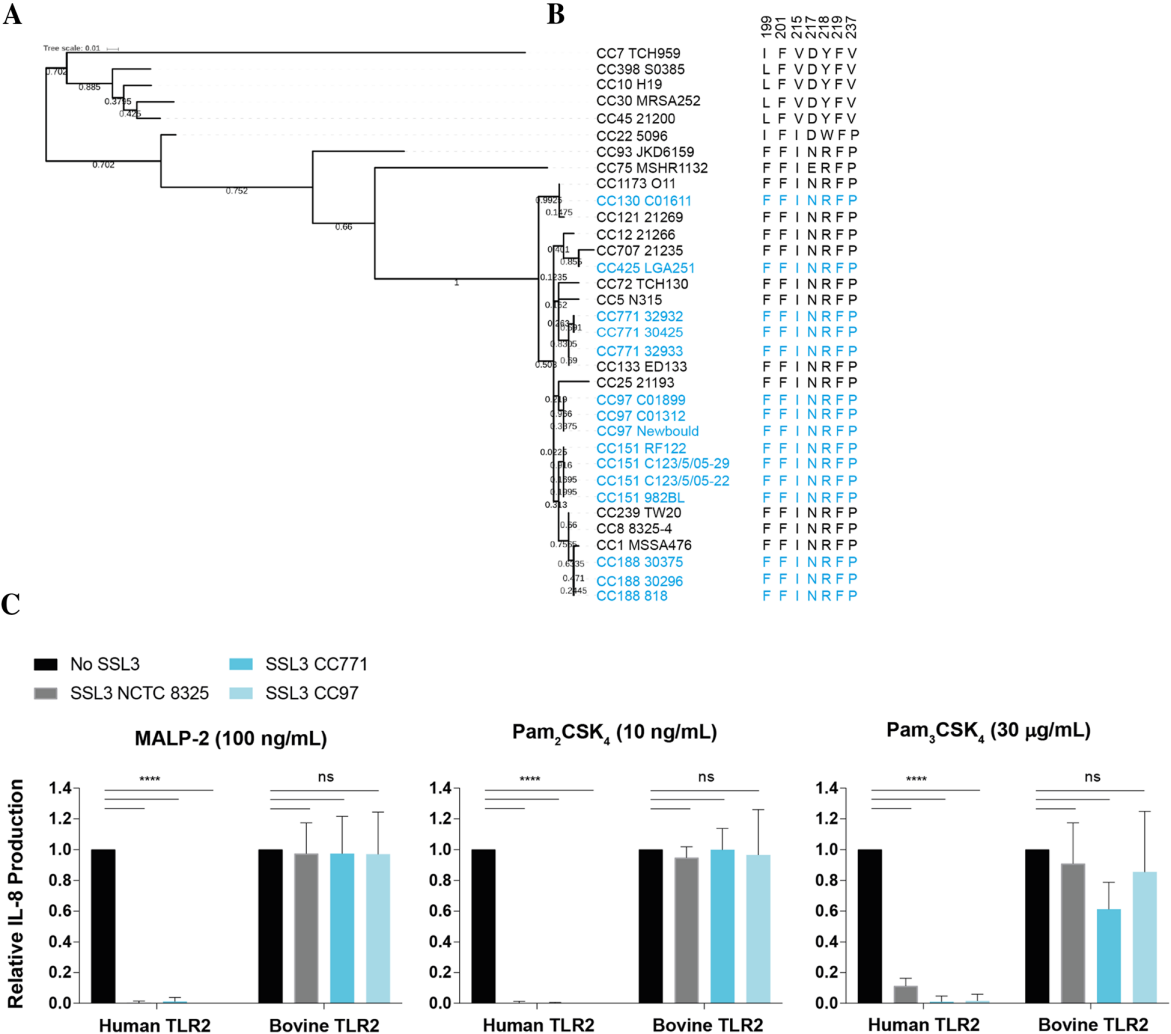
**SSL3 protein sequences from human and bovine strains show no signs of host adaptation. A** To investigate potential host adaptation of the SSL3 protein, the protein sequences from 19 human and animal (non-bovine) strains and 15 bovine strains, that represent the major lineages, were aligned and subsequently a maximum likelihood tree was produced in MEGA v6.05. Bovine strains are indicated in blue and non-bovine strains in black. **B** Shows the variation in SSL3 amino acids that were previously found to be essential for TLR2 inhibition. This region is highly conserved between all strains, with the exception of strains clustering with MRSA252, which was previously described to differ in this region [6, 8]. **C** HEK-TLR2 cells expressing human or bovine TLR2 were stimulated for 6 h with MALP-2 (100 ng/mL), Pam_2_CSK_4_ (10 ng/mL), or Pam_3_CSK_4_ (30 μg/mL) in presence or absence of three different SSL3 variants (from NCTC 8325, CC771, and CC97) in a final concentration of 10 μg/mL. IL-8 production was measured in ELISA. Data points represent the relative IL-8 production based on the situation without SSL3 present (set on 1.0 for both human and bovine cell lines) and the mean ± SD of three to four independent experiments is shown. Statistical analysis was performed using a one-way ANOVA followed by Tukey’s multiple comparison test to compare between groups. Statistical significance is indicated for the SSL3 treated cells as compared to the non-treated cells. ****p ≤ 0.0001, ns (non-significant).

Many immune evasion proteins function in only a narrow range of hosts, and different variants of the same molecules have been demonstrated to possess different host specificities. Such host-adapted variants are typically found at high frequency in *S. aureus* lineages associated with that specific host [2]. We investigated whether the major bovine *S. aureus* lineages (e.g. CC151, CC97, CC771) contained unique SSL3 variants absent in human and other animal *S. aureus* lineages (Additional file 3). Figure 5A shows the evolutionary relatedness of SSL3 molecules from 15 different bovine strains (shown in blue) belonging to six different clonal complexes (CC), and the SSL3 molecules from major human and other animal *S. aureus* lineages (shown in black). The analysis demonstrated that bovine *S. aureus* isolates do not possess a unique SSL3 variant, but instead showed that the SSL3 molecules of bovine strains cluster with those of multiple non-bovine lineages. Furthermore, though the SSL3 molecule is variable, the residues responsible for the TLR2 interaction are fully conserved amongst all bovine strains and the majority of other lineages (Figure 5B). The only differences found at the TLR2 interface occured in six lineages (CC7, CC10, CC22, CC45, CC30, and CC398) including the SSL3 molecule from MRSA252. This SSL3 variant was earlier described to contain a lowered activity due to amino acid differences in the specific region involved in TLR2 inhibition [6, 8]. Thus, we found no evidence that bovine *S. aureus* isolates possess SSL3 molecules that have adapted to the bovine host.

To confirm the suspected lack of host adaptation for SSL3, we cloned and expressed two SSL3 variants found in major bovine *S. aureus* strains: CC771 and CC97. These variants were subsequently examined for their ability to inhibit both human and bovine TLR2 (Figure 5C). Both variants were able to effectively inhibit human TLR2 after stimulation with the three agonists, alike to SSL3 from strain NCTC 8325 (CC8). Similarly, all three SSL3s could not inhibit stimulation of bovine TLR2. This confirms the suspected lack of host adaptation in SSL3 variants found in strains from bovine origin.

**Figure 6:**
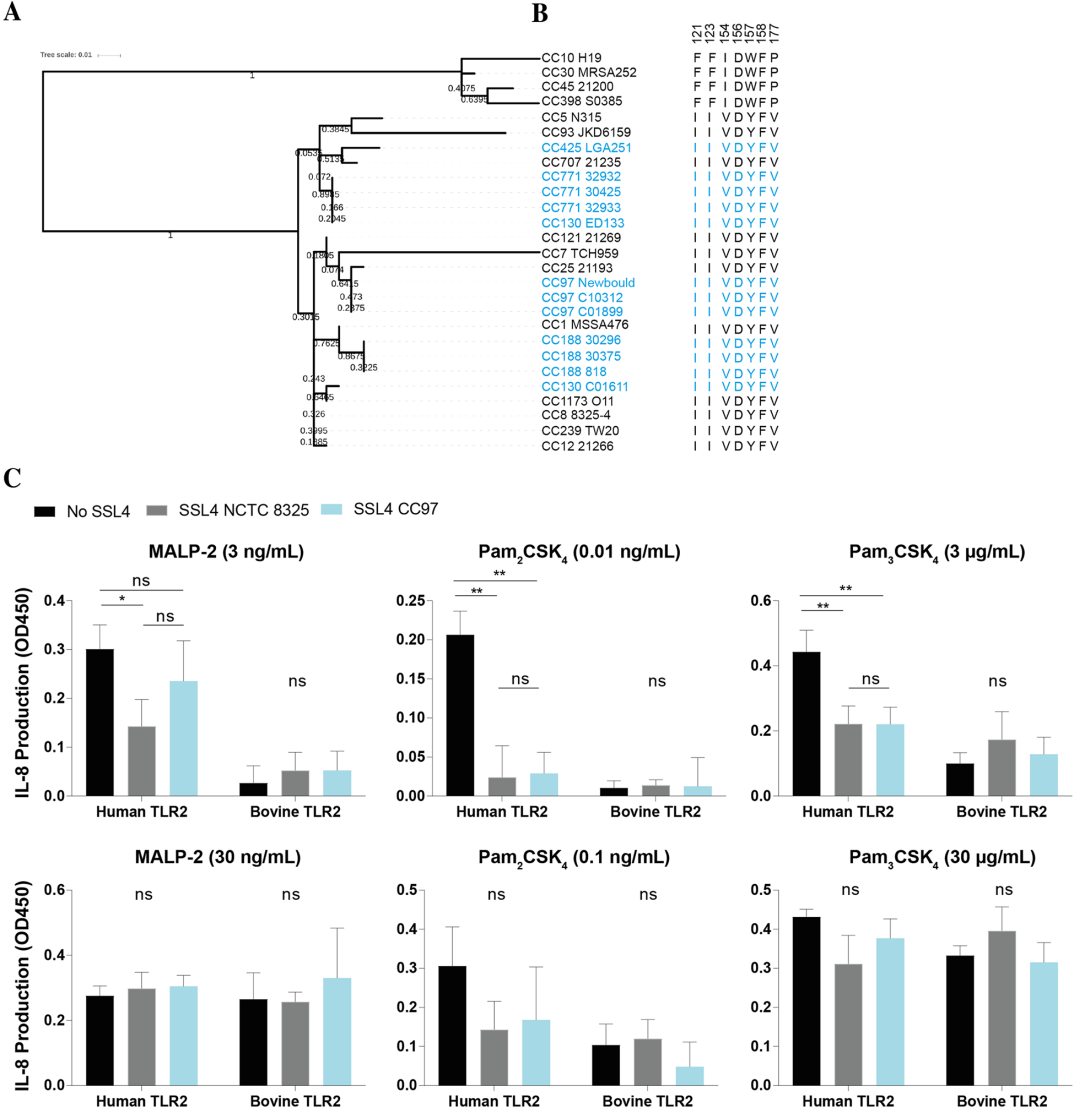
**SSL4 protein sequences from human and bovine strains show no signs of host adaptation. A** To investigate potential host adaptation of the SSL4 protein, the protein sequences from 15 human and animal (non-bovine) strains and 12 bovine strains, that represent the major lineages, were aligned and subsequently a maximum likelihood tree was produced in MEGA v6.05. Bovine strains are indicated in blue and non-bovine strains in black. **B** Shows the variation in SSL4 amino acids that were previously found to be essential for TLR2 inhibition. This region is highly conserved between all strains, with the exception of strains clustering with MRSA252, which was previously described to differ in this region [6, 8]. **C** HEK-TLR2 cells expressing human or bovine TLR2 were stimulated for 6 h with a low and high concentration of MALP-2 (3 ng/mL or 30 ng/mL), Pam_2_CSK_4_ (0.01 ng/mL or 0.1 ng/mL), or Pam_3_CSK_4_ (3 μg/mL or 30 μg/mL) in presence or absence of two different SSL4 variants (from NCTC 8325 and CC97) in a final concentration of 10 μg/mL. IL-8 production was measured in subsequent ELISA and data points represent the actual IL-8 production (OD_450_). The mean ± SD of at least three independent experiments is shown. Statistical analysis was performed using a one-way ANOVA followed by Tukey’s multiple comparison test to compare between groups. *p ≤ 0.05, **p ≤ 0.01, ns (non-significant).

As previously mentioned and shown in Additional file 1, SSL4 is an alternate less efficient inhibitor for human TLR2 and, as expected, the tested SSL4 from NCTC 8325 does not function on bovine TLR2. Alignments of SSL4 variants found in bovine strains (Figure 6 and Additional file 4) also showed no indication towards the existence of a host-adapted SSL4 variant, with all previously identified essential amino acids in SSL4 conserved between strains from both non-bovine and bovine origin. To confirm this, SSL4 from bovine strain CC771 was expressed and examined for TLR2-inhibitory capacity. Both SSL4 from NCTC 8325 (CC8) and CC771 were able to inhibit human TLR2 after stimulation with a low concentration of TLR2 agonist (Figure 6). Using higher agonist concentrations SSL4 is no longer able to inhibit TLR2, probably due to its lower binding-affinity as compared to SSL3. Activation of bovine TLR2 was not seen at these low agonist concentrations (Figures 2B–D, 6) and inhibition by SSL4 on bovine TLR2 could also not be measured at higher agonist concentrations. Together this indicates that the SSL4 variants found in bovine strains also do not possess activity towards bovine TLR2 and do not show any signs of host adaptation. Neither SSL3 nor SSL4 molecules from bovine strains demonstrated host-adaptation. Previously, *ssl* genes have been reported to be under the influence of purifying selection [26], which could act to maintain SSL functions. A codon-based test of neutrality DNA sequences revealed that neither *ssl3* and *ssl4* genes were neutrally evolving (*p* < 0.05 for both *ssl3* and *ssl4* datasets). Instead, a model of strict neutrality was rejected in favor of codon-based purifying selection (*p* < 0.05 for both *ssl3* and *ssl4*). This suggests that selective constraints are acting to maintain the SSL3 and SSL4 functions, and may suggest that different variants of SSL3 and SSL4 possess different functions.

## Discussion

In this study we have investigated the molecular basis for the SSL3–TLR2 interaction from the TLR2 perspective. We previously identified seven amino acids in SSL3 involved in the targeting of TLR2, but on the TLR2 side the molecular basis remained to be elucidated. Our study revealed two tyrosine residues in TLR2 that play an important role in the SSL3–TLR2 interaction: Tyr326 and Tyr376, with Tyr376 contributing most to the inhibitory potency of SSL3 to inhibit TLR2. Tyr376 being the most crucial residue determining SSL3 activity on TLR2 is in line with its direct interactions with two phenylalanine residues in SSL3 that were found essential for SSL3 function (Phe156 and Phe158) [8]. The identification of residue Tyr376 within TLR2 as the critical residue confirms that this region of the contact surface is of crucial importance for the SSL3–TLR2 interaction. These results also explain the species specificity of the SSL3 molecule: we found that bovine TLR2, which lacks the above mentioned tyrosine residues, is not inhibited by SSL3, whereas human, murine, and equine TLR2 are inhibited.

From sequence alignments we can predict whether TLR2 from other species will or will not be inhibited by SSL3. Rat and rabbit TLR2 will most likely be inhibited in a similar fashion as human and murine TLR2. Porcine TLR2 only differs in Tyr323, for which no essential role was observed in our experiments, and will therefore probably also be potently inhibited by SSL3. Sheep TLR2 does not possess Tyr376 and therefore is probably not efficiently inhibited by SSL3, and goat TLR2 contains the exact same residues as bovine TLR2 and thus will most likely also not be inhibited by SSL3. Thus, members of the ruminant family appear to be protected from SSL3-mediated immune evasion. The results also explain why equine TLR2 is efficiently inhibited by SSL3 (it contains residue Tyr376), but the lack of Tyr326 might result in the slight difference in SSL3 affinity as well as activity, observed after stimulation with Pam_2_CSK_4_ (Figure 3B).

*Staphylococcus aureus* is highly adaptive, and many strains have evolved to colonize and/or cause infection in humans or certain specific animal species. Immune evasion proteins have been shown to play a major, if not determining, role in host adaptation and the capacity of *S. aureus* to switch hosts [2]. *S. aureus* has recently (estimated 100–1000 years ago) jumped from humans to cattle, and has become bovine-adapted through a combination of gene loss, gene diversification, and acquisition of mobile genetic elements encoding virulence proteins with attenuated or enhanced function in the bovine host [27]. A prominent example of this host adaptation is the phage-encoded leukocidin LukMF’ that has enhanced toxic activity on bovine cells and is only found in bovine *S. aureus* lineages [28]. The immune evasion molecules encoded on the core variable genome typically have a broader host-specificity than mobile genetic element encoded immune evasion molecules. Nonetheless, allelic variants of core variable genome encoded immune evasion proteins have been demonstrated to possess different functions, which suggests that they may have important roles in host adaptation. It is surprising that even though SSL3 does not function on bovine TLR2, it is still present in the major *S. aureus* bovine isolates (e.g. CC151, CC97, CC771). Other studies have also demonstrated that the majority of bovine *S. aureus* carry the *ssl3* gene [29, 30]. In addition, the SSL3 molecule found in the major bovine lineages did not possess differences in the amino acids that were previously determined to be important for SSL3 function (Figure 5 and Additional file 3) and when these protein variants were expressed and examined for their inhibitory potential the proteins did not show inhibitory activity towards bovine TLR2, thus showing no signs of host adaptation on SSL3. Collectively, this could suggest that SSL3 possesses an undiscovered function in cattle. In support, the *ssl* genes have evolved under purifying selection that acts to maintain SSL functions and this suggests different variants may possess different functions. Furthermore, many SSLs have previously been shown to have multiple binding partners and functions [2], and *ssl3* is the most variable gene amongst the *ssl* genes [21]. Otherwise it is surprising that *S. aureus* lineages specifically colonizing and infecting ruminants would carry *ssl3*, while their hosts are not affected by it. It could be that circumvention of TLR2 signaling in the udder, where most *S. aureus* infections in these species occur (mastitis), is not important for bacterial survival. Another explanation could be that *S. aureus* has developed an alternative method in the cow to prevent effective TLR2 signaling. Mastitis caused by *S. aureus* has shown to result in very moderate host responses through limited TLR signaling, as reviewed previously [31], which is related to the well-known ability of *S. aureus* to cause chronic intramammary infections. This points towards staphylococci having alternative ways to circumvent TLR2-mediated immune responses in the bovine system.

Overall, ruminants appear to have evolutionary divergence in the specific area in TLR2 that is also involved in SSL3 binding. Since this region of TLR2 is involved in heterodimerization and ligand binding it is possible that TLR2 dimerization and ligand recognition differs in ruminants as compared to other species. Previous studies have reported on species-specific recognition of lipopeptides [24, 32, 33]. In the HEK cell lines used in this study, only the TLR2 of each species was recombinantly expressed. The dimerization partners (TLR1 and TLR6) are present at low endogenous levels on the HEK cells and are all of human origin [24]. Interestingly, all TLR2 s could be activated by the tested ligands (MALP-2, Pam_2_CSK_4_, and Pam_3_CSK_4_). Thus, it appears that TLR2 s from all species tested can form functionally active heterodimers with human TLR1 and TLR6 and that the endogenous receptor expression levels on HEKs are sufficiently high for stimulation. An alternative explanation would be that there is formation of functional TLR2 homodimers. This has been proposed before, but reports on this have been varying and the general consensus reached is that TLR2 homodimerization does not result in functional signaling [16, 17]. It is remarkable that bovine TLR2 was less efficiently stimulated by all tested agonists. These species-specific differences in signaling could be explained by TLR2 itself or the interaction with its dimerization partners, TLR1 and TLR6. One of the three investigated tyrosine residues in this study, Tyr326 in TLR2, is involved in ligand binding [17]. The substitution found in the cow (a histidine, Tyr326His) might result in changes in lipopeptide binding. This same position is described to be polymorphic in cattle and is in some cases occupied by a glutamine residue [34]. It remains to be determined how and if these amino acids in TLR2 affect lipopeptide recognition. The observed differences may also be due to the combination of bovine TLR2 with human TLR1 or TLR6. It has been previously described that triacylated lipopeptides do not stimulate bovine TLR2 well without the presence of the bovine TLR1 counterpart [24]. Since we expressed bovine TLR2 in a human TLR1 background this could explain why less efficient stimulation with Pam_3_CSK_4_ was observed. Similarly, this could also be the case for diacylated lipopeptides and TLR2/6. Inter-species differences exist in the dimerization region of TLR2–TLR6 [18], and this could explain suboptimal interactions between dimerization partners from different species. Altogether, amino acid differences in either TLR1, TLR2, or TLR6 could affect ligand binding and/or dimerization, which could result in species-specific TLR activation. Investigation of SSL3 function in a full bovine system (for example, primary bovine cells or a bovine cell line) was hindered by (likely nonspecific) activation of these cells by contaminants in the SSL3 preparations. The SSL3 used in our studies is purified in *E. coli* and thus likely contains remnants of LPS, which even in low concentrations will result in cell activation through TLR4. An effective way to circumvent TLR4-mediated activation via LPS is to use a combination of Polymyxin B and a TLR4 blocking mAb [6]. Unfortunately, such a blocking TLR4 mAb is not available for the bovine system and usage of only Polymyxin B could not prevent activation of the cells (nor can it for human primary cells). Thus, the development of more bovine-specific tools would be required to perform robust analysis in a bovine system and limits our study to the more purified system using HEK cell lines.

Structural and mutagenesis studies are valuable in revealing the molecular basis of an interaction as they can predict and identify amino acids involved in protein–protein interactions. This can be useful in the development of novel therapeutics, where knowing the exact foundation of the molecular interaction is essential. Revealing the residues in TLR2 that are targeted by SSL3 is critical for developing a strategy to interfere with the interaction, for example through SSL3-based derivatives such as peptides. Furthermore, this study contributes to the elucidation of the species specificity of immune evasive strategies of bacteria. The species specificity of virulence molecules is directly related to the host range of staphylococcal infections. Moreover, when developing therapeutics based on virulence molecules the species range of these molecules needs to be evaluated for translational purposes. It also underscores the importance of a proper choice of animal model when studying virulence factors. A therapeutic approach directed against SSL3 might be useful in the human system, but would likely not aid to prevent staphylococcal infections in cows (e.g. vaccination against bovine mastitis) or, most likely, in any other member of the ruminant family. Taken together, the current study reveals the molecular basis for the targeting of TLR2 by SSL3 and explains the species specificity of the interaction, thereby providing insights into staphylococcal host specificity.

## Additional files

**Additional file 1.** **SSL4 is not active on bovine TLR2.** HEK cells stably expressing bovine TLR2 were treated with different concentrations of SSL4 (ranging from 30 μg/mL to 1 μg/mL) before addition of different concentration of Pam_2_CSK_4_ (ranging from 300 ng/mL to 3 ng/mL). Supernatant was harvested after 6 h and IL-8 production was measured using an anti-IL-8 ELISA. One representative experiment is shown.

**Additional file 2.** **Expression of the different mutant TLR2 receptor cell lines.** (A) TLR2-FLAG expression of all mutant TLR2s was confirmed with anti-FLAG staining using Flow Cytometry. (B) HEK293T cell lines expressing all mutant TLR2s (human shown in black and bovine shown in blue) were stimulated with a concentration range of Pam_2_CSK_4_ for 6 h before harvesting of supernatants and subsequent IL-8 ELISA. Data points represent mean plus SD (errors bars shown above data points) of at least three independent experiments.

**Additional file 3.** **Multiple sequence alignment of SSL3 sequences.** The alignment shows SSL3 sequences from 34 *S. aureus* strains. The clonal complex (CC) of each strain is shown. Bovine and non-bovine strains are colored blue and black, respectively. Positions of residues required for forming the SSL3–TLR2 interface are shown with a red asterix. Sequences were aligned with ClustalW multiple alignment tool, and the alignment was colored using Jalview 2.1 according to the amount of sequence conservation (% Identity), in which a dark color indicates high sequence identity.

**Additional file 4.** **Multiple sequence alignment of SSL4 sequences.** The alignment shows SSL4 sequences from 27 *S. aureus* strains. The clonal complex (CC) of each strain is shown. Bovine and non-bovine strains are colored blue and black, respectively. Positions of residues required for forming the SSL4-TLR2 interface are shown with a red asterix. Sequences were aligned with ClustalW multiple alignment tool, and the alignment was colored using Jalview 2.1 according to the amount of sequence conservation (% Identity), in which a dark color indicates high sequence identity.
