# Supplementary figures and images for: Molecular basis determining species specificity for TLR2 inhibition by staphylococcal superantigen-like protein 3 (SSL3)
Source: Vet Res. 2018 Nov 28;49:115. doi: 10.1186/s13567-018-0609-8 (PMC6263051; doi:10.1186/s13567-018-0609-8)

**
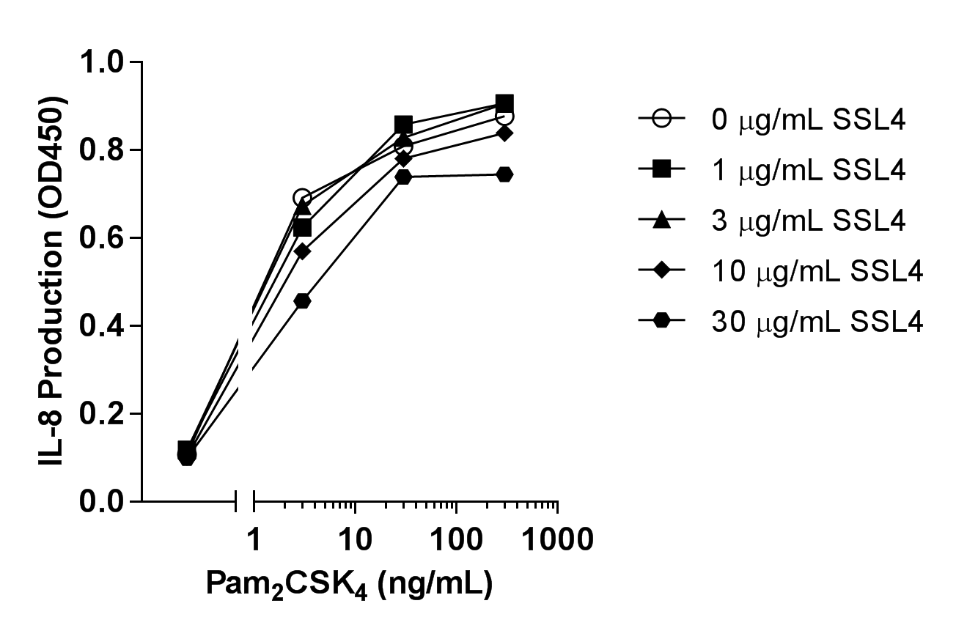
**

Supplement: Supplementary file 1 — Additional file 1. SSL4 is not active on bovine TLR2. HEK cells stably expressing bovine TLR2 were treated with different concentrations of SSL4 (ranging from 30 μg/mL to 1 μg/mL) before addition of different concentration of Pam2CSK4 (ranging from 300 ng/mL to 3 ng/mL). Supernatant was harvested after 6 h and IL-8 production was measured using an anti-IL-8 ELISA. One representative experiment is shown. [file 13567_2018_609_MOESM1_ESM.docx]

**
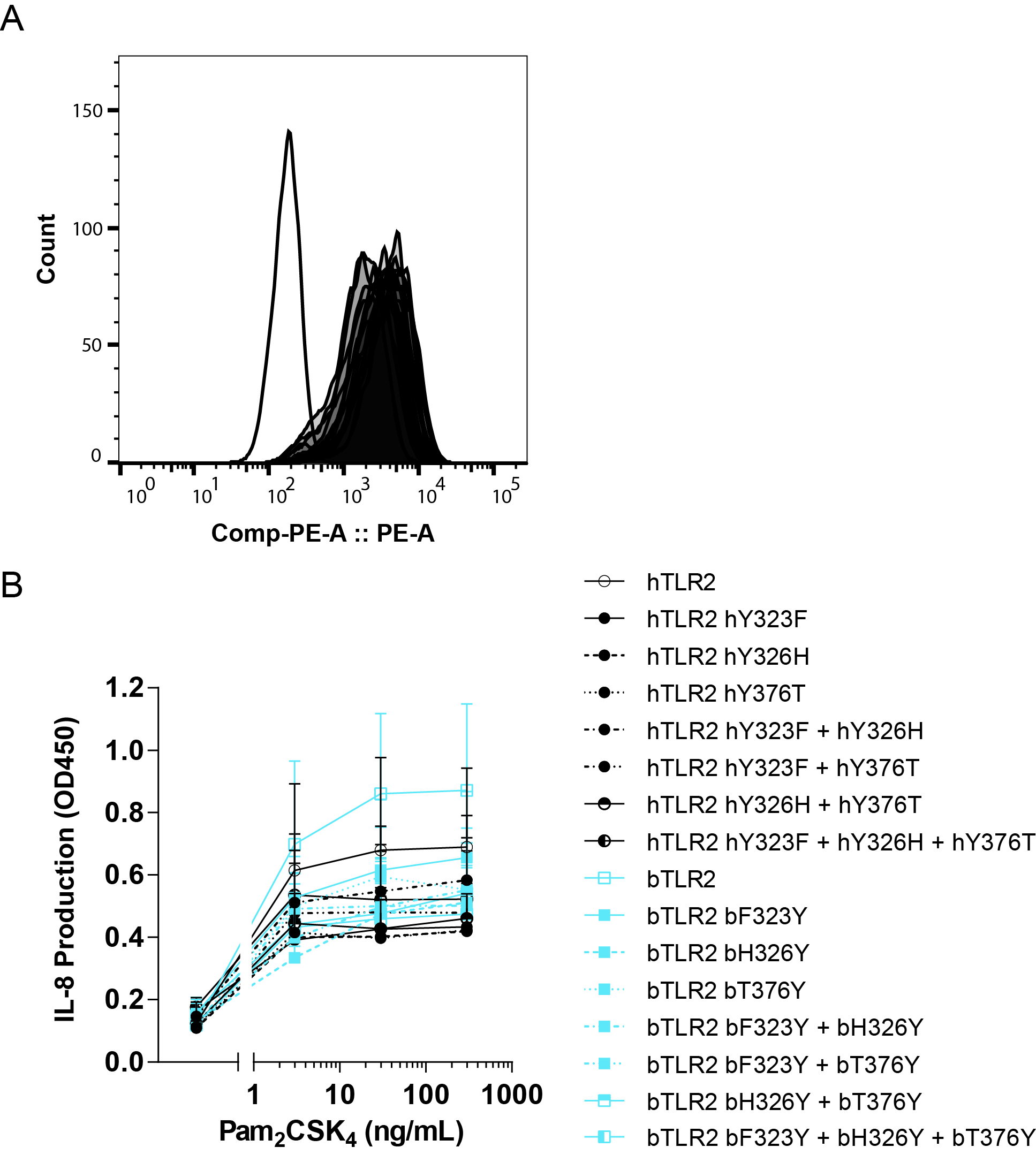
**

Supplement: Supplementary file 2 — Additional file 2. Expression of the different mutant TLR2 receptor cell lines. (A) TLR2-FLAG expression of all mutant TLR2s was confirmed with anti-FLAG staining using Flow Cytometry. (B) HEK293T cell lines expressing all mutant TLR2s (human shown in black and bovine shown in blue) were stimulated with a concentration range of Pam2CSK4 for 6 h before harvesting of supernatants and subsequent IL-8 ELISA. Data points represent mean plus SD (errors bars shown above data points) of at least three independent experiments. [file 13567_2018_609_MOESM2_ESM.docx]

**Additional file 3**


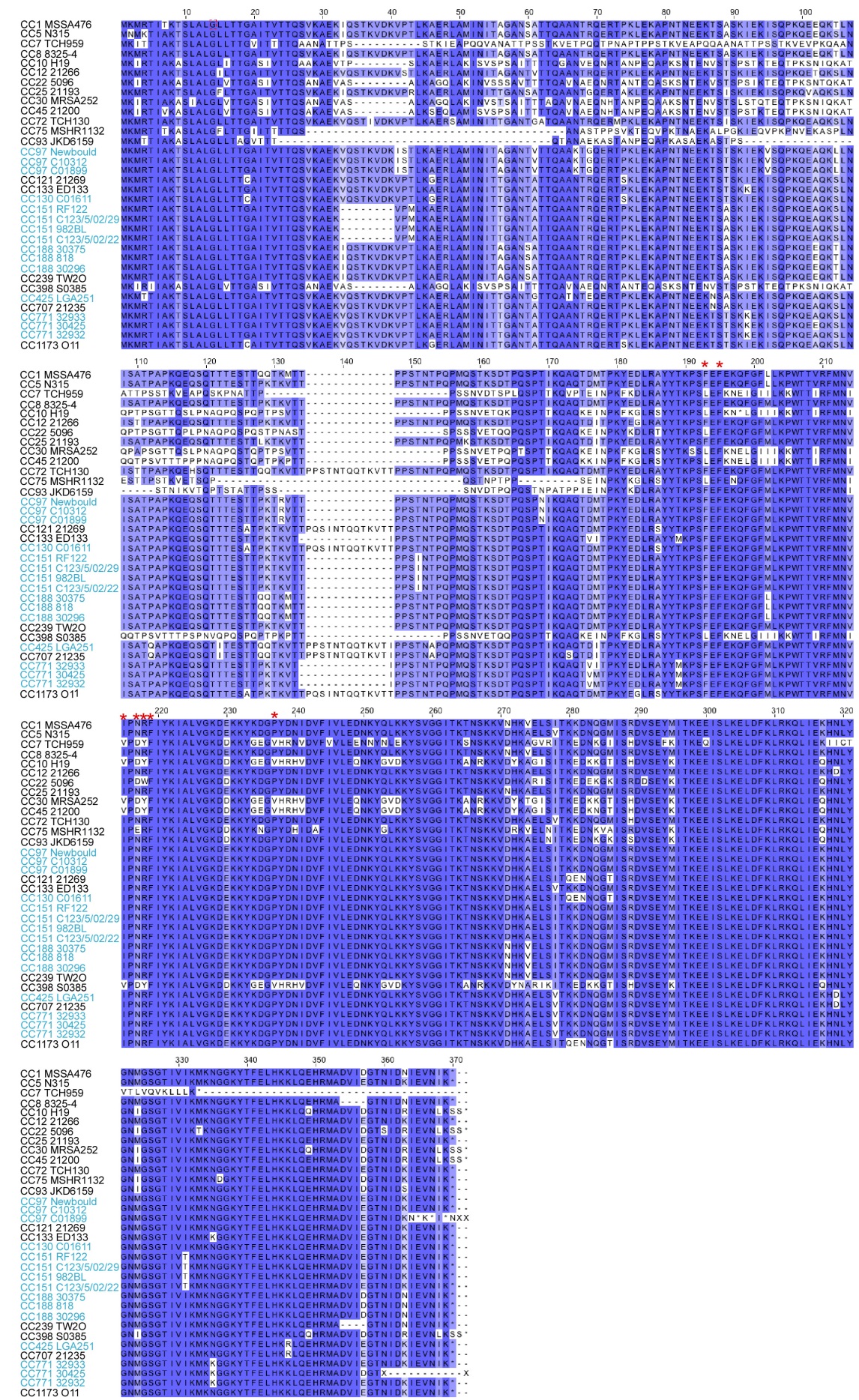

Supplement: Supplementary file 3 — Additional file 3. Multiple sequence alignment of SSL3 sequences. The alignment shows SSL3 sequences from 34 S. aureus strains. The clonal complex (CC) of each strain is shown. Bovine and non-bovine strains are colored blue and black, respectively. Positions of residues required for forming the SSL3–TLR2 interface are shown with a red asterix. Sequences were aligned with ClustalW multiple alignment tool, and the alignment was colored using Jalview 2.1 according to the amount of sequence conservation (% Identity), in which a dark color indicates high sequence identity. [file 13567_2018_609_MOESM3_ESM.docx]

**Additional file 4**


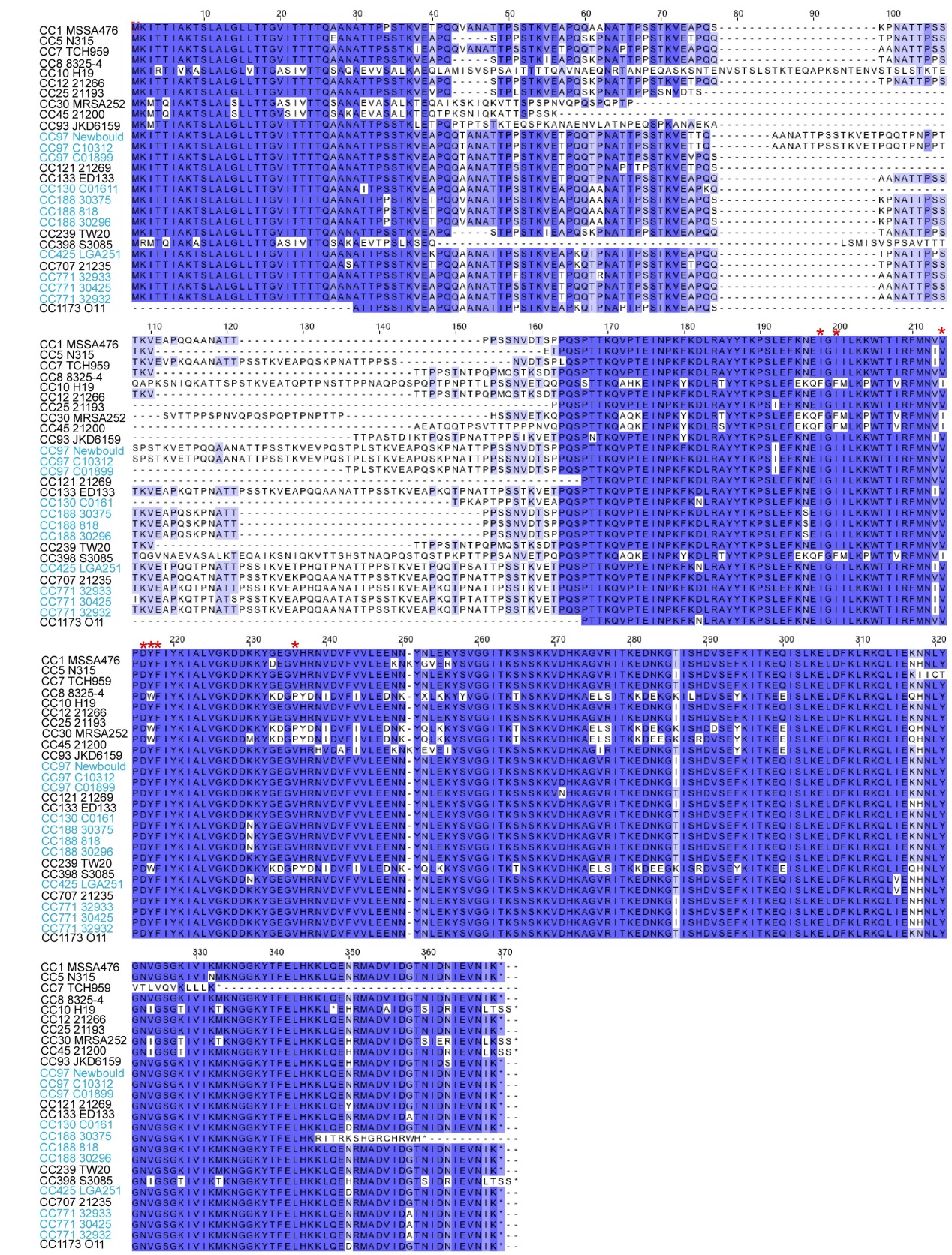

Supplement: Supplementary file 4 — Additional file 4. Multiple sequence alignment of SSL4 sequences. The alignment shows SSL4 sequences from 27 S. aureus strains. The clonal complex (CC) of each strain is shown. Bovine and non-bovine strains are colored blue and black, respectively. Positions of residues required for forming the SSL4-TLR2 interface are shown with a red asterix. Sequences were aligned with ClustalW multiple alignment tool, and the alignment was colored using Jalview 2.1 according to the amount of sequence conservation (% Identity), in which a dark color indicates high sequence identity. [file 13567_2018_609_MOESM4_ESM.docx]
